# The Evolution of Thermoplastic Raw Materials in High-Speed FFF/FDM 3D Printing Era: Challenges and Opportunities

**DOI:** 10.3390/ma18061220

**Published:** 2025-03-09

**Authors:** Antreas Kantaros, Meropi Katsantoni, Theodore Ganetsos, Nicolae Petrescu

**Affiliations:** 1 Department of Industrial Design and Production Engineering, University of West Attica, 12244 Athens, Greece; 2 Doctoral School of Political Sciences, Faculty of Political Sciences, University of Bucharest, 050107 Bucharest, Romania

**Keywords:** high-speed 3D printing, thermoplastic materials, FFF/FDM technology, additive manufacturing, PLA, ABS, PETG, mechanical properties, dimensional accuracy, advanced motion control systems

## Abstract

The evolution of thermoplastic materials has played a critical role in advancing high-speed Fused Filament Fabrication (FFF) and Fused Deposition Modeling (FDM) 3D printing technologies. This study explores the performance and challenges associated with next-generation thermoplastics specifically designed for high-speed printing, such as high-speed PLA, ABS, and PETG, in comparison to conventional materials. A systematic analysis was conducted to evaluate the key parameters, including the mechanical properties, layer adhesion, surface finish, and dimensional accuracy, under varying high-speed printing conditions. The results reveal that high-speed thermoplastics, when coupled with advanced hardware and optimized motion control systems, achieve up to a 70% reduction in printing time without significant trade-offs in mechanical integrity or precision. Additionally, the study identifies challenges, such as increased thermal stresses, warping, and the need for precise cooling strategies, which can impact material performance at elevated speeds. Opportunities for future development are also discussed, including the design of novel polymer formulations and hardware innovations to further enhance the reliability and scalability of high-speed FFF/FDM printing. This work underscores the potential of adopting such advanced thermoplastic materials in the high-speed 3D printing era and highlights the critical interplay between material science and hardware engineering for achieving next-generation manufacturing capabilities.

## 1. Introduction

**Figure 1:**
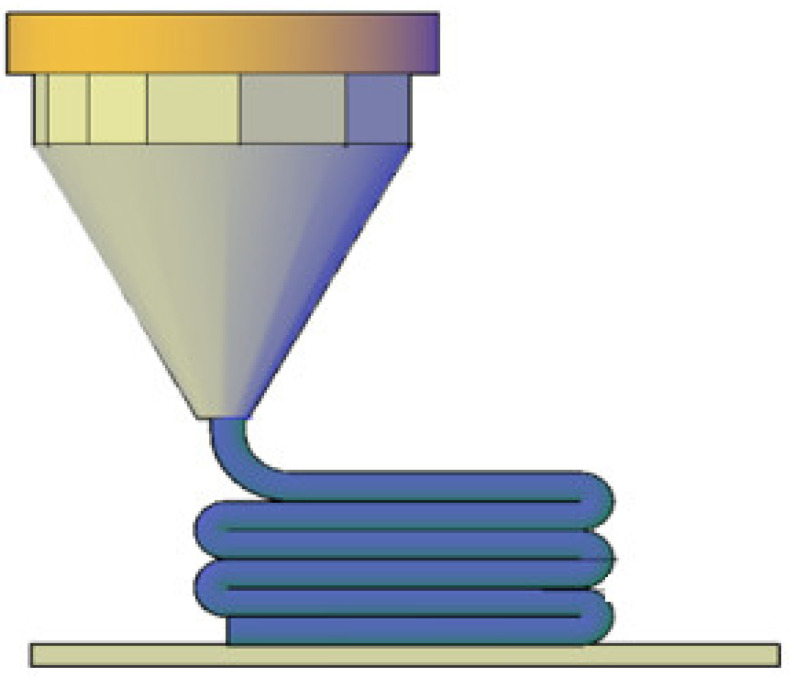
Schematic representation of a heated nozzle in an FFF/FDM 3D printer, depositing the molten thermoplastic material on a layer-by-layer modus operandi.

Three-dimensional printing, also known as additive manufacturing, has rapidly evolved from a prototyping technology to a versatile manufacturing tool across various industries, including aerospace, automotive, healthcare, and consumer products [1]. Fused Filament Fabrication (FFF) and Fused Deposition Modeling (FDM) have gained significant popularity due to their accessibility, ease of use, and broad adoption across home, semi-professional, and industrial applications. However, they are not the only additive manufacturing technologies available. Other well-established techniques, such as Stereolithography (SLA), Digital Light Processing (DLP), Liquid Crystal Display (LCD) printing, bioprinting, Material Jetting, and Powder Bed Fusion, offer distinct advantages depending on the application requirements. Despite these alternatives, FFF/FDM remains the most widely used method due to its cost-effectiveness and versatility. Therefore, this study focuses on the evolution of thermoplastic materials specifically within the high-speed FFF/FDM 3D printing domain, highlighting the advancements and challenges unique to this technology. The materials used in 3D printing are a crucial element, influencing not only the performance of the printed objects but also the overall efficiency, cost, and scalability of the printing process [2]. Among the various material types employed in 3D printing, thermoplastics have gained widespread use due to their favorable properties, such as ease of processing, mechanical strength, and adaptability to different printing techniques [3,4]. Thermoplastic materials are polymers that can be repeatedly softened by heating and hardened by cooling, allowing them to be reshaped without undergoing chemical degradation. This characteristic is particularly beneficial for 3D printing as it enables the creation of complex detailed geometries with relatively low energy input. Figure 1 depicts a schematic representation of a heated nozzle in an FFF/FDM 3D printer, depositing the molten thermoplastic material on a layer-by-layer modus operandi.

Common 3D printing thermoplastics include PLA, ABS, and PETG. PLA is user-friendly, biodegradable, and prints at low temperatures. ABS offers durability, impact resistance, and heat tolerance for functional parts [5,6,7,8]. PETG combines ABS’s strength with PLA’s ease of printing, ensuring good adhesion and chemical resistance. Other materials like PC, nylon, and TPU provide flexibility, strength, or wear resistance, serving industries such as automotive and medical manufacturing [9,10,11,12]. The continued advancement in 3D printing technologies has led to the development of new thermoplastic formulations that are designed to address specific industry needs, such as high-temperature resistance, improved print speeds, and enhanced mechanical properties [13,14,15].

The evolution of 3D printing technologies has not only been marked by advancements in materials but also by significant improvements in the printers themselves. High-speed 3D printers have emerged as a response to the increasing demand for faster production times, particularly in industries requiring rapid prototyping and small-scale manufacturing [16,17]. These printers are designed to offer significantly higher print speeds compared to traditional 3D printers, enabling faster layer deposition and reducing the overall print times [18]. Manufacturers such as Bambu Labs (Shenzhen, China) [19], Creality (Shenzhen, China) [20], and Prusa (Prague, Czech Republic) [21] have introduced high-speed 3D printing desktop equipment, incorporating advanced features that limit the time needed for printing operations to be completed.

**Figure 2:**
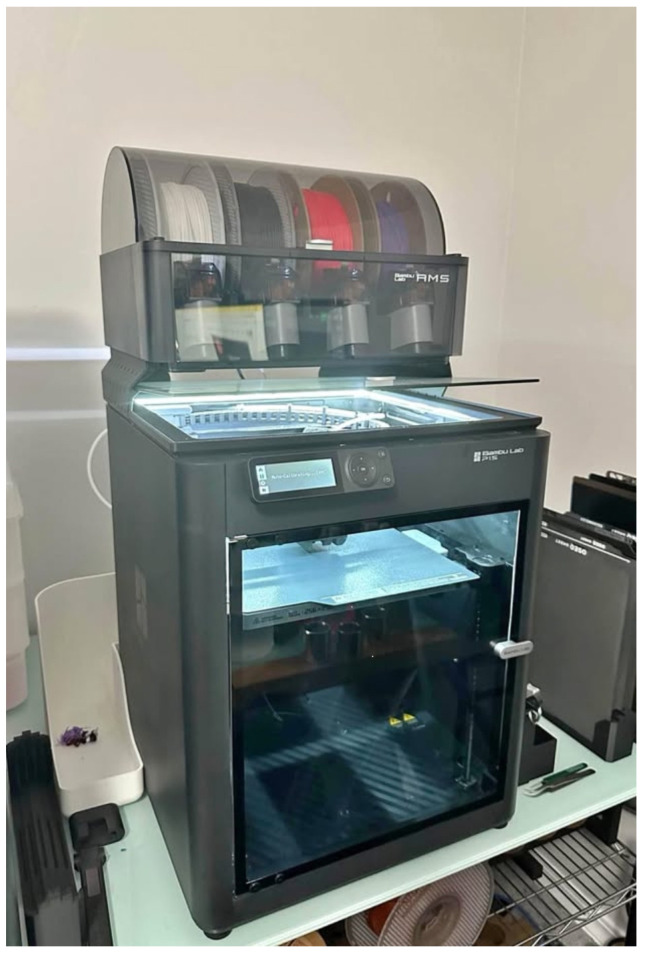
Bambu Labs P1S enclosed 3D printer.

Bambu Labs (Shenzhen, China), for instance, has gained attention for its integration of multi-material and multi-color printing capabilities, coupled with high-speed performance, making it a versatile choice for professional and commercial use [22]. While a normal speed for the established desktop FFF/FDM category would be in the range of 50–70 mm/s, these 3D printers exhibited a maximum deposition speed of 600 mm/s, with an average speed of 300 mm/sec being sufficient to attribute even the most intricate design details. The company’s high-speed printers leverage an innovative system that combines advanced hardware with optimized slicing software, allowing for faster printing without compromising quality [23]. Similarly, Creality (Shenzhen, China), a well-established name in the 3D printing community, has introduced high-speed models like the K1 series, which offer improved print speeds, enhanced reliability, and ease of use [24]. These printers incorporate features such as upgraded extruders and faster stepper motors, along with improved hotends that allow for higher throughput. Prusa (Prague, Czech Republic), known for its precision and reliability, has also introduced high-speed printing options with the Prusa MK4 model, which boasts improved hardware and software optimizations to achieve faster print speeds without sacrificing the quality of finished prints [25,26]. These advancements in speed are accompanied by developments in enhanced cooling systems and print beds, as well as specialized hotends, allowing for smoother extrusion at higher speeds. Figure 2 depicts a Bambu Labs P1S enclosed 3D printer.

The push for higher printing speeds is not just about reducing the time it takes to produce a part; it also ties into the growing need for greater efficiency in industries that rely on additive manufacturing. High-speed printers enable quicker iterations in proto-typing, faster production times in low-volume manufacturing, and the ability to meet tight production deadlines [27]. As such, they represent a shift toward more commercial-grade 3D printing, offering a viable alternative to traditional manufacturing processes. However, these advancements come with their own set of challenges, including the need for more precise calibration, more expensive hardware, and potentially greater material costs.

The need for faster 3D printing has become increasingly critical across a range of industries, driven by the demand for quick turnaround times in product development, prototyping, and small-scale manufacturing. Traditional manufacturing processes, such as injection molding or machining, often require long lead times for tool changes, setup, and production, limiting their flexibility, particularly for custom or low-volume production. In contrast, 3D printing provides a faster and more cost-effective solution for creating complex geometries without the need for molds or specialized tooling. As industries such as aerospace, automotive, healthcare, and consumer goods continue to embrace additive manufacturing, the ability to produce parts and prototypes in significantly reduced timeframes has become a competitive advantage. Rapid prototyping, for instance, allows for iterative design cycles, enabling engineers and designers to quickly test and refine their concepts. High-speed 3D printers facilitate this process by drastically cutting down the production times per part, which is crucial for maintaining a fast pace of innovation and meeting tight market demands.

Moreover, the increasing adoption of mass customization in industries like healthcare, fashion, and consumer electronics has further amplified the need for faster 3D printing technologies. In healthcare, for example, the ability to produce patient-specific implants, prosthetics, and anatomical models on demand has revolutionized personalized medicine [28,29,30,31]. However, to ensure that these custom products meet the rapid needs of patients, healthcare providers require faster and more efficient production methods. The same holds true for the fashion industry, where designers seek to create unique on-demand products without the constraints of traditional manufacturing processes. Additionally, industries such as automotive and aerospace are exploring additive manufacturing for spare parts and low-volume production runs, where speed is essential to reduce downtime and operational costs [32]. As the demand for faster and more cost-effective solutions in these sectors continues to rise, the development of high-speed 3D printing technologies becomes increasingly important. These advancements not only reduce the time to market but also enable industries to achieve more flexible on-demand production capabilities, which is essential in an era where agility and rapid response to customer needs are paramount.

Thus, the primary purpose of this paper is to conduct a comparative analysis be-tween newly developed high-speed thermoplastic materials and their older counterparts, focusing on their respective properties, performance characteristics, and suitability for various applications. As high-speed 3D printing technologies continue to evolve, the introduction of materials that are specifically engineered to optimize performance at faster print speeds presents an important area of study. By examining the differences in their mechanical properties, such as strength, flexibility, and thermal resistance, as well as the printability of these materials, this paper aims to provide a comprehensive understanding of how these new materials perform under the high-speed printing conditions facilitated by advanced printers like those from Bambu Labs (Shenzhen, China), Creality (Shenzhen, China), and Prusa (Prague, Czech Republic). Additionally, the paper will analyze the trade-offs associated with adopting these high-speed materials, particularly in terms of material cost, availability, and potential compromises in part quality, such as surface finish or dimensional accuracy. The implications of these materials on industrial practices will also be explored, considering how they influence production speed, cost-effectiveness, and the ability to meet market demands for rapid prototyping and on-demand manufacturing. By synthesizing data from both current high-speed materials and traditional thermoplastics, this paper seeks to provide insights into the potential for these new materials to revolutionize 3D printing applications across diverse sectors while also addressing the challenges that may arise from their adoption in professional and commercial environments.

## 2. Background and Evolution of Thermoplastic Materials for 3D Printing

The early days of 3D printing were primarily defined by the use of a limited set of thermoplastic materials that provided a starting point for the development of the industry. Among the most widely used were polylactic acid (PLA), acrylonitrile butadiene styrene (ABS), and polyethylene terephthalate glycol (PETG), each offering distinct advantages that made them suitable for different applications. PLA, one of the first materials extensively used in 3D printing, is a biodegradable polymer derived from renewable resources such as corn starch or sugarcane [33]. Its popularity in the early stages of 3D printing stemmed from its ease of use, low printing temperature (typically around 190–220 °C), and non-toxic properties, making it ideal for hobbyists, educators, and beginners. PLA’s relatively low thermal expansion also made it suitable for achieving dimensional accuracy in printed parts, especially for simple geometries and prototyping. However, while PLA offered advantages in ease of use, it had limitations in terms of heat resistance and mechanical strength, which made it unsuitable for high-performance or functional parts that required durability under stress or high temperatures [34].

ABS, another foundational material in 3D printing, quickly gained favor due to its enhanced durability, toughness, and impact resistance compared to PLA. ABS is a petroleum-based polymer that requires a higher printing temperature (approximately 220–250 °C) and typically needs a heated bed to prevent warping during the cooling process [35]. These characteristics made ABS an attractive choice for engineers and designers who needed to produce more robust parts for applications that involved mechanical stress or exposure to higher temperatures. ABS’s greater flexibility and strength, combined with its ability to be easily post-processed (e.g., through sanding or acetone vapor smoothing), made it a staple in the early stages of 3D printing, particularly for industries like automotive, where the parts required both strength and reliability [36]. However, ABS also had some drawbacks, including its tendency to emit potentially harmful fumes during printing, requiring good ventilation in printing environments, and its susceptibility to warping, which could affect the quality and dimensional accuracy of large prints [37].

PETG, which emerged as a later alternative, offered a balanced solution that combined some of the best properties of both PLA and ABS [38]. PETG is a glycol-modified version of PET, a material commonly used for making plastic bottles, and it offers enhanced durability, chemical resistance, and impact strength compared to PLA. It is also easier to print with than ABS, with a similar printing temperature range of 220–250 °C, but without the need for a heated bed in many cases. PETG is known for its superior layer adhesion, reducing the risk of warping during the cooling process, and it produces prints with a smooth, glossy finish that is aesthetically pleasing [39]. As such, it gained popularity for applications that required both functionality and visual appeal, such as in consumer goods, packaging, and electronics. Additionally, PETG’s chemical resistance makes it suitable for parts exposed to moisture, oils, or chemicals, further expanding its versatility. While PETG addressed some of the limitations of both PLA and ABS, it still faced challenges related to printing speed and its tendency to string or ooze, which affected the precision of certain print jobs [40].

Over the past decade, material science has played a pivotal role in the rapid expansion and evolution of 3D printing technologies. A key advancement has been the development of specialty thermoplastics and composite materials that offer enhanced performance characteristics for specific applications. For example, the introduction of high-performance polymers such as polycarbonate (PC) [41], nylon [42], and polyether-etherketone (PEEK) [43] has significantly broadened the range of industrial applications for 3D printing. Polycarbonate, known for its high impact resistance and optical clarity, has become a popular material in industries such as aerospace and automotive, where strength and transparency are crucial. Similarly, nylon’s high tensile strength, flexibility, and resistance to wear and tear make it ideal for producing functional prototypes, end-use parts, and even medical devices. PEEK, one of the highest-performing thermoplastics in terms of temperature resistance, mechanical strength, and chemical inertness, has found its place in highly demanding industries such as aerospace, medical implants, and high-end manufacturing. These materials, once difficult to print with due to their high melting points and material-specific challenges, have been optimized through advancements in 3D printing equipment, such as the development of specialized heated print beds, high-temperature extruders, and precision control systems.

In addition to pure polymers, the development of composite materials for 3D printing has revolutionized the possibilities for creating stronger, lighter, and more functional parts [44]. Composites that integrate fibers such as carbon fiber, glass fiber, and even metal particles into thermoplastics have gained traction in industries that require parts with superior mechanical properties [45,46,47]. Carbon-fiber-reinforced filaments, for example, of-fer remarkable stiffness and strength-to-weight ratios, making them ideal for applications in aerospace, automotive, and robotics [48]. The introduction of metal-filled filaments has also expanded the potential for 3D printing to create parts with enhanced heat resistance, electrical conductivity, and corrosion resistance, particularly in the manufacturing of functional tools, prototypes, and end-use parts [49]. Moreover, advancements in the formulation of flexible materials, such as thermoplastic polyurethane (TPU), have addressed the growing demand for soft, elastomeric parts in industries such as healthcare, footwear, and automotive [50]. These innovations in material science have significantly improved the mechanical, thermal, and chemical properties of 3D-printed parts, enabled more di-verse applications, and expanded the scope of 3D printing beyond prototyping and into full-scale manufacturing.

As mentioned in the previous chapter, the introduction of high-speed 3D printing technology has significantly altered the landscape of additive manufacturing, driving not only improvements in printing hardware but also spurring innovations in the development of new materials. High-speed 3D printers, which utilize advanced features such as faster extrusion rates, improved motion systems, and optimized slicing algorithms, require materials that can be processed efficiently without compromising the quality of the printed parts [50]. This has led to the emergence of specialized high-speed filaments that are designed to flow more smoothly through extruders, cure faster under heat, and maintain their mechanical integrity when printed at higher speeds. As a result, material developers have focused on optimizing existing polymers and creating entirely new formulations that can withstand the demands of rapid printing processes while maintaining the desired properties, such as strength, flexibility, and durability [51]. These new materials often incorporate additives or reinforcements that enhance their printability, such as improved flowability, faster cooling times, and better adhesion between layers, all of which are crucial for achieving the precision and performance expected in high-speed 3D printing.

In parallel, the need for high-speed materials has accelerated the development of filaments with enhanced thermal stability and dimensional accuracy. High-speed 3D printing often involves extrusion at higher temperatures, which can induce issues like warping, stringing, and surface defects when using standard materials. To address these challenges, material scientists have formulated high-speed variants of traditional thermoplastics, such as high-speed PLA, ABS, and PETG, which feature optimized molecular structures and additives that reduce the risk of warping and improve the material’s ability to handle rapid cooling rates [52]. High-speed PLA, for instance, is engineered with a modified polymer blend that retains the ease of printing associated with standard PLA but offers improved layer bonding and reduced cooling times, making it more suitable for high-speed applications [53]. Similarly, high-speed ABS and PETG formulations are designed with faster crystallization rates and greater thermal stability, allowing for consistent part quality even when printed at accelerated speeds [54]. These advancements not only improve the overall performance of high-speed 3D printers but also expand the range of potential applications for additive manufacturing, enabling faster production of more durable and functional parts across industries such as automotive, aerospace, and healthcare. In this context, Table 1 summarizes the key thermoplastic materials used in 3D printing, highlighting their properties, typical applications, and the impact of high-speed 3D printing on their use.

## 3. New High-Speed 3D Printing Raw Materials

As mentioned before, with the introduction of high-speed 3D printers, a demand for new materials capable of performing efficiently under rapid printing conditions has been created. In this chapter, the high-speed variants of commonly used thermoplastics, such as PLA, ABS, and PETG, will be examined, focusing on the enhancements made to optimize their performance in faster printing environments. The chemical compositions and structural modifications that distinguish these materials from traditional filaments will be explored, along with the claims made by manufacturers like Bambu Labs (Shenzhen, China) regarding their specific formulations. The typical applications and industries where these high-speed materials are being adopted to improve production processes and reduce turnaround times will also be discussed.

**Figure 3:**
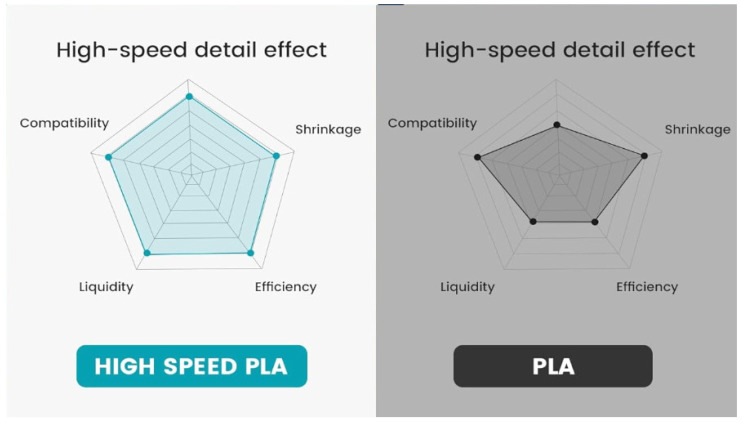
Differences in exhibited properties of regular and high-speed PLA when subjected to high printing speeds [57].

High-speed PLA is an evolution of the traditional PLA filament, designed to optimize performance when used with high-speed 3D printers. High-speed PLA has been engineered with modified polymer blends that offer faster curing times and enhanced layer adhesion, allowing for smoother prints at higher speeds [55]. This material often incorporates additives that improve its flowability and reduce cooling times, which are essential for high-speed printing processes. The improvements in high-speed PLA make it a viable option for rapid prototyping, where time to market is a critical factor. While it retains most of the benefits of traditional PLA, such as low environmental impact due to its biodegradability, it also addresses the need for faster production cycles in applications that do not require extreme heat resistance or mechanical strength [56]. High-speed PLA finds extensive use in industries where rapid prototyping, low-cost production, and detail precision are paramount, such as consumer goods, educational tools, and decorative items. Figure 3 depicts the differences in exhibited properties of regular and high-speed PLA when subjected to high printing speeds, as presented by the well-known 3D printing raw material manufacturer SUNLU [57].

More specifically, the manufacturer states that the material demonstrates superior print quality compared to traditional PLA at normal print speeds, with a smoother surface and improved overhang performance. Overhang angles of up to 70° can be achieved without the need for supports, significantly reducing material waste and printing time. The viscosity of high-speed PLA is reduced by 500%, enhancing its melt flow characteristics and ensuring consistent extrusion without clogging, even at speeds exceeding 250 mm/s. Additionally, the improved thermal conductivity facilitates efficient heat dissipation, promoting rapid cooling and better layer adhesion during the printing process. These optimized rheological properties contribute to superior extrusion performance, reducing the risk of under-extrusion and material shortages at high printing speeds [57]. High-speed PLA also boasts a high crystallization temperature, enabling rapid curing and excellent drape performance, with the ability to print without supports for overhang angles under 70°. The material’s low shrinkage rate further reduces the likelihood of cracking during fast printing, resulting in a smooth finish. Furthermore, the filament is designed for consistency, with uniform wire diameter that ensures even, smooth extrusion without knotting, contributing to efficient and reliable high-speed printing [57].

On the other hand, high-speed ABS, developed as a direct response to the limitations of traditional ABS, is designed for applications that demand higher mechanical strength, temperature resistance, and durability. Standard ABS requires careful temperature control and the use of a heated bed to prevent warping and ensure optimal layer adhesion, especially in larger prints. High-speed ABS variants are formulated with additives that enhance the material’s processing capabilities at faster extrusion rates. These formulations maintain the material’s essential qualities, such as toughness and impact resistance, but are more suited to high-speed printing environments [58]. The main benefit of high-speed ABS is its ability to produce strong, durable parts with a reduced print time, which is crucial for industries such as automotive and manufacturing, where the rapid production of functional prototypes and parts is necessary. While high-speed ABS reduces printing time, challenges such as warping and post-processing (sanding or smoothing) still per-sist, and users must ensure appropriate environmental controls (e.g., heated build plat-forms or enclosed print areas) to achieve optimal results [59]. Despite these challenges, high-speed ABS is increasingly being used in applications that demand functional performance, such as automotive prototypes, tools, and housings for electronics.

**Figure 4:**
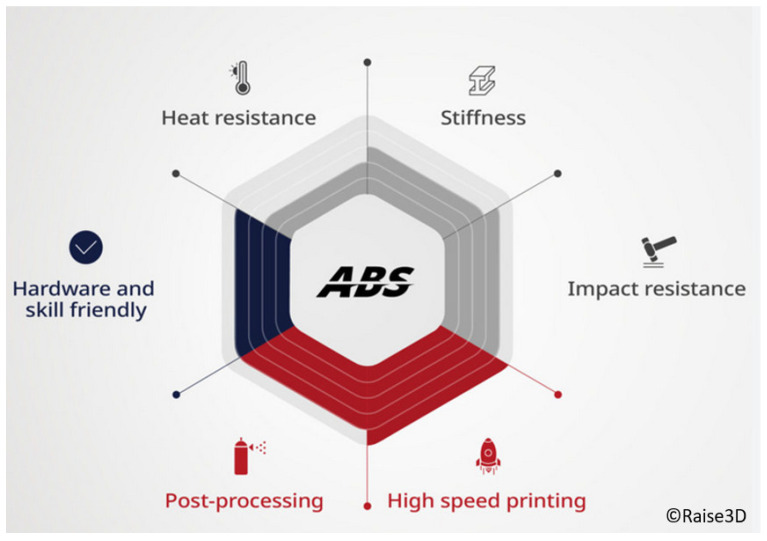
Differences in exhibited properties of regular and high-speed ABS when subjected to high printing speeds [60].

Well-known 3D printer and raw material manufacturer Raise3D (Shenzhen, China) mention that their high-speed ABS, called “Hyper Speed ABS” filament, is designed specifically for high-speed 3D printing, particularly when used with the Hyper FFF^®^ solution from the Hyper Speed filament line [60]. This filament addresses the common challenges of high-speed printing with standard ABS, such as nozzle clogging, poor layer bonding, warping, and delamination, by offering optimized molecular weight and stiffness. This filament addresses the common challenges of high-speed printing with standard ABS, such as nozzle clogging, poor layer bonding, warping, and delamination, by offering optimized molecular weight and stiffness. Specifically, the molecular weight is reduced compared to standard ABS, enhancing melt flow characteristics, improving extrusion stability, and minimizing the risk of nozzle clogging while maintaining sufficient mechanical strength and interlayer adhesion. The material’s faster melting and more uniform cooling allow it to be printed at speeds of up to 300 mm/s, minimizing temperature gradients and internal stresses, thus in turn reducing warping and enhancing interlayer bonding. Hyper Speed ABS also boasts superior heat resistance (up to 77 °C, HDT@0.45 MPa), excellent impact resistance, and greater dimensional stability, making it ideal for functional prototypes, tools, and fittings. Furthermore, it is well suited for post-processing techniques like polishing and acetone vapor smoothing, adding versatility to its applications [60]. In this context, Figure 4 depicts a graph illustrating the differences in exhibited properties of regular and high-speed ABS when subjected to high printing speeds.

Likewise, high-speed PETG is a modification of the commonly used PETG filament, designed to meet the growing demand for faster print speeds while retaining the material’s well-regarded characteristics. PETG is known for its excellent strength, impact resistance, and chemical resistance, but traditional PETG can be challenging to print at high speeds due to its tendency to string and ooze. High-speed PETG, however, has been formulated with specialized additives that reduce these issues and improve its flow characteristics, enabling smoother and faster printing without sacrificing material integrity [61]. The faster crystallization rates of high-speed PETG allow it to cool more efficiently, reducing the potential for warping and improving layer adhesion. Additionally, the material’s ability to maintain strength and durability at higher print speeds makes it particularly useful in industries where both performance and rapid production are required. This includes applications in packaging, medical devices, and consumer products, where the material’s combination of mechanical strength, chemical resistance, and ease of processing is highly valued. While high-speed PETG may still present challenges, such as the need for precise extrusion temperature control to avoid issues with over- or under-extrusion, its ability to handle faster printing without significant quality degradation has made it a preferred choice for industrial applications that require rapid prototyping and functional part production.

In this context, the well-known manufacturer Bambu Labs (Shenzhen, China) has introduced a range of high-speed 3D printing materials specifically formulated to address the challenges associated with rapid printing. Their TPU 95A HF filament, for example, is designed to be printed up to three times faster than standard TPU 95A, significantly reducing printing times without compromising quality. Additionally, the company’s High Performance Filament collection, which includes materials such as carbon-fiber-infused filaments, is optimized for high-speed printing applications and compatible with their AMS system [62]. Bambu Labs’ filaments feature enhanced properties, such as faster melting, improved layer bonding, and reduced warping, enabling efficient printing at speeds of up to 300mm/s. These materials cater to a variety of functional prototyping and manufacturing needs, offering improved heat resistance, impact resistance, and dimensional stability. The manufacturer’s filament guide further supports users by providing comprehensive information on material selection, ensuring that specific printing requirements and performance characteristics are met [62].

High-speed 3D printing materials can contribute to various industries by enabling rapid prototyping, efficient production, and the creation of complex geometries. In the automotive sector, manufacturers utilize these materials to produce lightweight components, functional prototypes, and tooling, thereby reducing development times and costs. For instance, Lotus has incorporated 3D printing technologies into their design process for the Theory 1 supercar, aiming to enhance performance and reduce weight through advanced manufacturing techniques [63].

In the medical field, high-speed 3D printing facilitates the creation of customized prosthetics, dental aligners, and surgical models, allowing for personalized patient care and improved surgical planning. The ability to rapidly produce patient-specific models aids in complex surgeries, enhancing precision and outcomes [64]. Likewise, the aero-space industry benefits from high-speed 3D printing materials by enabling the rapid pro-duction of lightweight complex parts, which are essential for improving fuel efficiency and performance. Additionally, these materials are employed in the creation of molds and tooling, streamlining the manufacturing process and reducing lead times [65]. Similarly, in the defense sector, high-speed 3D printing materials are utilized to produce durable and lightweight components for military equipment, enhancing performance and reducing weight. The ability to quickly manufacture parts on demand also improves maintenance and repair operations, ensuring the readiness of defense assets [65].

Overall, high-speed 3D printing materials are integral to various industries that had already adopted FFF/FDM 3D printing, offering benefits such as reduced production times, cost savings, and the ability to create complex customized parts that were previously time-consuming to fabricate.

## 4. Comparison of High Speed vs. Conventional Thermoplastic Raw Materials

The introduction of high-speed 3D printing materials has raised critical questions about how these new formulations compare to their traditional counterparts in terms of physical performance and practical usability. This chapter provides a detailed comparison of the mechanical properties, printability, performance at high speeds, thermal properties, and post-processing requirements of new high-speed materials versus older thermoplastics. By examining key attributes such as tensile strength, impact resistance, and flexibility, as well as factors like ease of printing and thermal behavior, the analysis aims to evaluate whether the advantages offered by these new materials justify their adoption in industrial and professional applications. Particular attention is paid to how these materials perform under the demanding conditions of high-speed printing and whether they maintain the reliability and quality required for functional parts.

### 4.1. Mechanical Properties

The mechanical properties of 3D printing materials play a critical role in determining their suitability for various applications, and the evolution of high-speed materials has introduced notable advancements in this domain. One of the most significant properties is tensile strength, which measures the material’s ability to resist breaking under tension. Traditional materials like standard PLA and ABS offer moderate tensile strength, making them suitable for prototypes and non-load-bearing applications [66]. However, high-speed variants, such as high-speed PLA and ABS, exhibit enhanced tensile properties due to optimized polymer chains and additives designed to improve interlayer adhesion. This results in stronger parts that can better withstand tensile loads, even when printed at high speeds [66]. These improvements make high-speed materials more suitable for functional parts where structural integrity is crucial, such as in automotive or aerospace applications [67].

Impact resistance, another key property, has undergone significant enhancements in high-speed 3D printing materials. Standard ABS, known for its relatively high impact resistance, often suffers from reduced layer bonding at high printing speeds, leading to weakened parts [68]. High-speed ABS addresses this limitation by incorporating additives and molecular modifications that improve energy absorption during impact. Similarly, high-speed PLA has been engineered to perform better under dynamic loads, offering a level of impact resistance that is closer to traditional ABS while retaining its ease of printing. These improvements enable the production of durable, impact-resistant parts, making high-speed materials more versatile for applications requiring both toughness and rapid manufacturing, such as tools, fixtures, and protective housings.

Flexibility is another crucial mechanical property, particularly for materials like TPU and nylon, which are often used in applications requiring elastic or semi-flexible parts. While standard TPU is known for its excellent elasticity, printing at high speeds can lead to inconsistent layer bonding and uneven extrusion, compromising the material’s mechanical performance [69]. High-speed TPU formulations have addressed this issue by enhancing the polymer’s flow characteristics and cooling properties, ensuring consistent flexibility even under rapid printing conditions [70]. Similarly, high-speed nylon has been developed to maintain its ductility and elongation-at-break properties, allowing for the creation of functional parts that require both strength and flexibility, such as hinges, gaskets, and vibration dampers [71].

Another critical aspect of mechanical properties is fatigue resistance, which deter-mines a material’s ability to endure repeated stress over time. Traditional thermoplastics like PLA and ABS often experience performance degradation under cyclic loading, especially when printed at lower layer heights or without optimized settings [72]. High-speed materials are designed to address this challenge through improved interlayer bonding and stress distribution, which reduce the likelihood of crack propagation and delamination [73]. These advancements are particularly valuable in industrial applications where parts are subjected to repetitive mechanical stress, such as robotics components or assembly line fixtures. Overall, the mechanical properties of high-speed 3D printing materials represent a significant leap forward, offering enhanced performance and durability with-out sacrificing the efficiency and speed that modern additive manufacturing demands.

### 4.2. Printability

Printability is a critical factor in evaluating 3D printing materials as it directly influences the efficiency, quality, and accessibility of the printing process. Traditional materials like PLA, ABS, and PETG have long been valued for their relatively user-friendly printing characteristics, but high-speed variants of these materials take printability to a new level. High-speed PLA, for instance, maintains the ease of printing associated with standard PLA but requires fewer adjustments to achieve optimal results at higher speeds. The im-proved flow characteristics of high-speed PLA allow it to extrude smoothly through the nozzle, minimizing clogging and enabling consistent layer deposition [74]. Additionally, the material’s reduced cooling times eliminate the need for extensive fine-tuning of print settings, such as fan speeds and retraction distances, which are often critical for avoiding stringing or warping in standard materials [75].

In the case of high-speed ABS, significant advancements have been made to address the challenges associated with its traditional counterpart, which typically requires a heated bed and enclosed build chamber to prevent warping and delamination [76]. High-speed ABS formulations are designed to reduce thermal expansion, allowing for more reliable prints at higher speeds without the need for strict environmental controls. This improvement makes the material more accessible for users who lack advanced 3D printers with heated chambers as the likelihood of failed prints due to warping is significantly reduced [77]. Furthermore, high-speed ABS demonstrates superior adhesion between layers, even at high extrusion speeds, which contributes to better dimensional accuracy and part strength [77]. These enhancements in printability make high-speed ABS a more practical choice for both professional and industrial applications, where rapid production cycles and functional part quality are paramount.

The printability of high-speed PETG has also been enhanced to address the limitations of standard PETG, which is prone to issues such as stringing, oozing, and inconsistent layer adhesion [78]. High-speed PETG formulations incorporate additives that im-prove the material’s thermal stability and viscosity, ensuring smoother extrusion and faster cooling [78]. These changes enable users to print at speeds of up to 300 mm/s while maintaining excellent layer adhesion and surface finish. Additionally, high-speed PETG is less sensitive to minor fluctuations in temperature and print settings, making it more forgiving for users with less precise printers or those operating in non-ideal conditions [79]. Such improved stability and ease of printing allow high-speed PETG to be used effectively in applications requiring both speed and strength, such as functional prototypes, packaging, and consumer products.

Another aspect of printability is the compatibility of high-speed materials with advanced printing systems and software. Many high-speed materials, such as those developed by manufacturer Bambu Labs (Shenzhen, China), are specifically optimized for use with high-performance 3D printers equipped with features like advanced motion systems, high-flow extruders, and precise cooling mechanisms. These printers enable the materials to achieve their full potential in terms of speed and quality. Moreover, slicing software tailored for high-speed printing ensures that the optimized print profiles for these materials are readily accessible, reducing the trial-and-error process often required with standard materials. The seamless integration of high-speed materials with cutting-edge hardware and software not only simplifies the printing process but also enhances overall productivity, making them indispensable tools for industries that rely on fast and reliable additive manufacturing.

### 4.3. Performance at High Speeds

The performance of high-speed 3D printing materials at rapid print speeds represents a significant advancement over traditional thermoplastics, enabling greater efficiency and precision in additive manufacturing. Traditional materials such as standard PLA, ABS, and PETG often struggle to maintain consistent mechanical properties and print quality when printing at high speeds due to insufficient time for the polymer to fully melt and adhere between layers. This results in defects such as poor layer bonding, warping, and surface irregularities [80]. High-speed materials overcome these challenges through modifications to their molecular structures, including enhanced flowability and faster crystallization rates. These changes allow the material to transition more effectively from a solid to a molten state, ensuring even deposition and superior interlayer adhesion even at extrusion speeds exceeding 250–300 mm/s. This improved performance at high speeds translates directly into higher productivity without compromising the quality of the printed parts [81].

**Figure 5:**
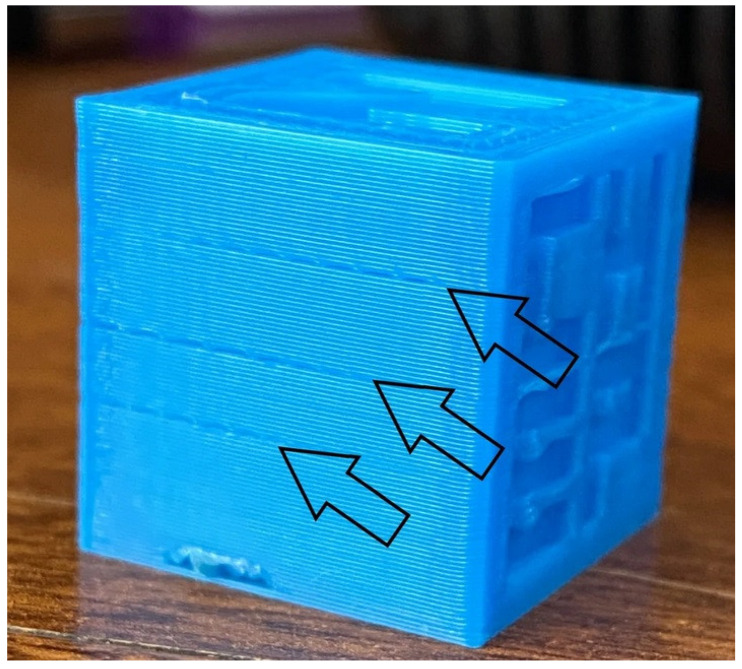
Dimensional inaccuracies and surface finish flaws (indicated by the black arrows) on a 3D-printed item fabricated with conventional PLA raw material.

One of the key metrics of material performance at high speeds is dimensional accuracy [82]. Traditional materials often suffer from dimensional distortions when printed rapidly due to the uneven cooling and thermal stresses introduced by faster printing. High-speed materials address this issue by incorporating stabilizers and additives that reduce thermal expansion and improve cooling behavior [83]. For instance, high-speed PLA and ABS exhibit a more uniform cooling process, reducing the risk of shrinkage and deformation. This enhanced thermal stability enables the production of parts with precise geometries and tight tolerances, even during high-speed printing [83,84]. Such improvements are particularly important in industries like aerospace and automotive, where exacting dimensional accuracy is critical for parts that need to fit within larger assemblies or perform under load. Figure 5 depicts the dimensional inaccuracies and surface finish flaws (indicated by the black arrows) on a 3D-printed item fabricated with conventional PLA raw material.

Another area where high-speed materials excel is in maintaining surface finish and detail resolution, even when printing at rapid speeds. Standard materials often produce rough surfaces or exhibit visible layer lines when extrusion rates are increased as the polymer lacks sufficient time to settle evenly between layers [85]. In contrast, high-speed materials are engineered to flow smoothly and solidify rapidly, resulting in parts with smoother surfaces and finer details even at high print speeds. This capability is critical for applications such as consumer products or medical devices, where both functionality and aesthetic quality are important [86]. Moreover, the reduction in post-processing requirements for high-speed materials, due to their ability to maintain surface quality, further enhances their appeal for rapid manufacturing [87].

Finally, high-speed materials demonstrate superior durability under the stresses im-posed by fast printing processes. Rapid printing can introduce additional dynamic stresses during extrusion and cooling, which can compromise the mechanical integrity of standard materials [88]. High-speed variants, however, are designed to withstand these conditions by enhancing layer adhesion and reducing the internal stresses that arise from rapid cooling. This durability ensures that the printed parts maintain their strength and reliability, even when produced at high speeds. Applications such as functional proto-types, tooling, and production-grade components benefit significantly from these performance enhancements as the parts are not only produced faster but also meet the mechanical and thermal requirements of demanding environments.

### 4.4. Thermal Properties

The thermal properties of 3D printing materials are critical to their performance, particularly for applications requiring durability under elevated temperatures or significant thermal stress [89]. One of the most important parameters is the glass transition temperature (Tg), which marks the point where the material transitions from a rigid state to a rubbery, more flexible one. For standard PLA, the Tg typically ranges between 55 °C and 65 °C, which limits its use in applications exposed to higher temperatures [90]. In contrast, high-speed PLA formulations often exhibit an elevated Tg due to modifications in their chemical structure, allowing them to retain their rigidity at higher operating temperatures [91]. These improvements make high-speed PLA more versatile, enabling its use in environments where standard PLA would deform, such as in automotive interiors or electronic housings exposed to heat.

Heat resistance is another critical property that has been significantly improved in high-speed thermoplastics. Traditional ABS, known for its moderate heat resistance, per-forms well up to approximately 100 °C but can suffer from warping and thermal expansion during the printing process [92]. High-speed ABS addresses these issues with optimized polymer chains and additives that enhance its thermal stability [93]. These advancements allow high-speed ABS to maintain its shape and structural integrity at elevated temperatures, making it ideal for functional prototypes, tooling, and parts subjected to heat during their service life [93]. Similarly, high-speed PETG has been engineered to offer superior heat resistance compared to its standard counterpart, enabling it to with-stand prolonged exposure to higher temperatures without compromising its chemical resistance or mechanical properties [94].

The thermal conductivity and cooling behavior of high-speed materials are also critical to their performance during printing [95]. Rapid printing introduces the need for materials that can dissipate heat efficiently to prevent overheating and ensure consistent lay-er deposition. High-speed filaments, such as those developed for advanced 3D printers, incorporate heat-dissipating additives that promote faster cooling. For example, high-speed PLA and PETG formulations have been optimized for rapid crystallization, enabling them to solidify more quickly and reduce the risk of print defects like sagging or surface inconsistencies [96]. This characteristic not only improves print quality but also contributes to faster production cycles as the material’s thermal properties are better aligned with the demands of high-speed extrusion and cooling systems.

Another significant thermal property is the material’s resistance to thermal cycling, which determines its ability to withstand repeated heating and cooling without degradation. High-speed materials are designed to exhibit minimal thermal expansion and con-traction, reducing the likelihood of cracks, delamination, or dimensional instability during printing or in service [97]. These properties are particularly valuable in applications such as aerospace, automotive, and medical devices, where parts may be subjected to fluctuating thermal environments. In this context, the combination of high glass transition temperatures, enhanced heat resistance, and improved thermal cycling performance makes high-speed materials more robust and reliable compared to traditional thermoplastics, expanding their usability in demanding industrial applications.

### 4.5. Post-Processing

The post-processing characteristics of high-speed 3D printing materials have been meticulously engineered to address the limitations associated with traditional thermoplastics, thereby enhancing their applicability in advanced manufacturing [98]. High-speed PLA, for instance, is designed to yield smoother surfaces immediately after printing due to its improved flow dynamics and rapid solidification properties. These modifications reduce the visibility of layer lines, often obviating the need for extensive sanding or polishing, a common requirement for standard PLA [99]. The superior surface finish of high-speed PLA significantly reduces post-processing time and effort, particularly in applications where aesthetic quality is paramount, such as consumer product prototypes and architectural models. Furthermore, the material’s compatibility with painting and coating processes is preserved, enabling high-quality finishes in functional and display-ready parts [100].

High-speed ABS exhibits advancements in post-processing efficiency through its enhanced structural uniformity and compatibility with chemical finishing methods, such as acetone vapor smoothing [101]. Standard ABS, while widely used for functional components, frequently requires intensive smoothing to achieve professional-grade surface finishes due to issues such as warping and uneven layer deposition. In contrast, high-speed ABS minimizes these defects by incorporating additives that optimize interlayer bonding and thermal stability, resulting in parts with inherently improved surface quality. These properties reduce the duration and intensity of vapor smoothing while also enhancing the structural integrity of the finished part [102]. Consequently, high-speed ABS is particularly suitable for applications demanding both high mechanical performance and refined aesthetics, such as automotive components, enclosures for electronic devices, and engineering prototypes.

High-speed PETG represents a further evolution in post-processing optimization by addressing common deficiencies in standard PETG, such as excessive stringing and sur-face inconsistencies [103]. The modified formulations of high-speed PETG improve its thermal stability and flow characteristics, leading to more uniform layer deposition and a smoother surface finish directly out of the printer. This improvement not only reduces the need for sanding or filing but also enhances the material’s compatibility with advanced finishing techniques, such as chemical treatments and precision coatings. These attributes make high-speed PETG an excellent choice for applications requiring a balance of chemical resistance, mechanical strength, and visual appeal, such as medical devices, laboratory equipment, and high-end consumer goods [103]. The enhanced surface quality of high-speed PETG also improves the adherence and uniformity of paints, enabling customized finishes for diverse end-use applications.

The durability of high-speed materials during post-processing also demonstrates substantial advancements over traditional thermoplastics. Mechanical post-processing techniques, such as drilling, sanding, and machining, often compromise the structural integrity of parts printed with standard materials due to weaker interlayer bonding and inconsistent material properties [104]. High-speed materials mitigate this issue through optimized polymer structures and enhanced interlayer adhesion, ensuring that parts retain their mechanical performance even after rigorous post-processing. This robustness is particularly critical for industrial applications, where post-processed components are subjected to functional stresses, such as those encountered in aerospace, automotive, and manufacturing tools [104]. As a result, high-speed materials offer a dual advantage: they reduce the complexity of post-processing while preserving or enhancing the functional integrity of finished parts, making them indispensable in high-performance and precision applications [104]. Table 2 summarizes the key differences between traditional thermoplastics and high-speed materials across mechanical properties, printability, performance at high speeds, thermal properties, and post-processing requirements.

## 5. Challenges and Future Directions

The development and adoption of high-speed 3D printing materials represent a significant milestone in additive manufacturing. However, as with any transformative technology, the widespread implementation of these materials comes with unique challenges that need to be addressed. This chapter explores the obstacles associated with scaling production, the technical limitations of high-speed printing, and the potential for future innovations in material science. Finally, it examines how the evolution of high-speed thermoplastics will shape key industries such as automotive, aerospace, and healthcare, setting the stage for broader adoption and transformative industrial applications.

With the increasing adoption of Fused Filament Fabrication (FFF) for producing composite structural parts, it is crucial to consider the environmental implications of these materials. The use of composite filaments, which combine thermoplastics with reinforcing fibers such as carbon fiber or glass fiber, introduces both opportunities and challenges from a sustainability standpoint [105,106,107,108,109,110]. On one hand, composites can enhance the performance and durability of printed parts, potentially reducing the need for frequent replacements and contributing to longer product lifecycles [111,112,113]. On the other hand, the environmental impact of composite materials remains a concern. The production of these filaments often relies on non-renewable resources, and the recycling of composite materials can be complex due to the difficulty in separating the plastic matrix from the reinforcing fibers [114,115,116]. Furthermore, post-consumer waste from printed composite parts may contribute to landfill accumulation as many of these materials are not biodegradable. However, advancements in material science, such as the development of biodegradable composites and the use of recycled materials in filament production, are promising steps toward mitigating these environmental challenges. As the FFF process continues to evolve, it is essential to incorporate eco-friendly practices, such as improving recycling techniques and exploring renewable resources, to minimize the ecological footprint of composite-based 3D printing. Scaling the production of high-speed thermoplastics to meet specific industrial demands poses several challenges, particularly in ensuring consistency and quality across large production volumes. High-speed materials require precise formulations that involve advanced chemical modifications, such as additives for improved flowability and rapid crystallization. Maintaining the uniformity of these formulations at scale can be difficult as even slight variations in molecular structure or composition can lead to inconsistent performance during printing. Furthermore, specialized production processes, such as advanced compounding and extrusion techniques, are necessary to manufacture these materials, which increases the cost and complexity of scaling. For smaller manufacturers or industries with limited budgets, this represents a significant barrier to adoption.

Another challenge in scaling lies in the supply chain for high-speed thermoplastics. The production of these materials often relies on specialty polymers and additives, some of which may be expensive or difficult to source in bulk quantities. Additionally, pro-duction facilities that are capable of handling high-speed material formulations are limited, further constraining the supply. These issues can lead to bottlenecks in material availability, particularly as the demand for high-speed printing continues to rise. Addressing these challenges will require investments in manufacturing infrastructure, process optimization, and the development of alternative formulations that are both cost-effective and scalable.

Despite the advancements in high-speed 3D printing materials, technical limitations remain, particularly in maintaining accuracy and detail resolution at high print speeds. High-speed printing processes inherently introduce dynamic stresses on the material during extrusion and deposition, which can lead to inaccuracies in fine details and geometries. This is especially problematic for intricate designs, such as lattice structures or parts with small tolerances, where precision is critical. Furthermore, the rapid cooling and solidification of high-speed materials, while beneficial for productivity, can sometimes result in internal stresses that compromise their dimensional accuracy or lead to warping.

Another technical challenge is the limited compatibility of high-speed materials with the existing printer hardware. Not all 3D printers are equipped with the advanced motion systems, high-flow extruders, or precise cooling mechanisms required to fully utilize these materials. As a result, users operating standard or older machines may experience suboptimal performance, limiting the widespread adoption of high-speed materials. Advances in printer technology and software optimization will be crucial to overcoming these technical barriers, ensuring that high-speed materials can be used effectively across a broader range of printing systems.

The future of high-speed thermoplastics lies in further innovations that enhance their performance, broaden their applications, and reduce their production costs. One promising avenue is the development of hybrid materials that combine the properties of multiple thermoplastics, such as the strength of ABS with the flexibility of TPU, enabling the creation of parts with multifunctional properties. Additionally, the integration of nanomaterials, such as graphene or carbon nanotubes, into high-speed thermoplastics could further improve their mechanical, thermal, and electrical properties, expanding their usability in advanced manufacturing.

Material customization is another area of potential innovation. By tailoring material formulations to specific applications, such as aerospace components requiring extreme thermal stability or medical devices demanding biocompatibility, high-speed materials can become more specialized and targeted. Advances in computational material science and machine learning are expected to play a significant role in this process, enabling faster and more accurate predictions of material behavior under various printing conditions. These innovations will not only enhance the performance of high-speed materials but also drive their adoption across diverse industries.

A crucial aspect of high-speed 3D printing materials that warrants further exploration is the influence of raw material parameters on interfacial adhesion and toughness. Interfacial adhesion plays a pivotal role in determining the overall performance of printed parts, especially when composite materials are used in the FFF/FDM process. The interactions between the matrix material and reinforcing fillers (such as carbon fiber or glass fiber) are critical for achieving optimal mechanical properties, including strength, impact resistance, and fracture toughness. Variations in raw material properties, such as the molecular structure, polymer chain alignment, and the compatibility of fillers, can significantly impact the degree of adhesion at the interface. This, in turn, affects the material’s overall toughness, particularly under stress or impact conditions. Microstructural factors, such as crystallinity, porosity, and the dispersion of reinforcing fibers, also play a significant role in fracture behavior as they influence how the material responds to applied loads. For example, materials with higher crystallinity typically exhibit better strength but may be more brittle, while materials with a higher degree of amorphousness may demonstrate better toughness but reduced strength. Additionally, processing variables such as print speed, extrusion temperature, and layer bonding contribute to the material’s final microstructure and, consequently, its fracture behavior. Consolidating the existing studies on these factors can offer valuable insights into how raw material properties and processing conditions affect the interfacial adhesion and fracture toughness of high-speed 3D printing materials, providing a more comprehensive understanding of their behavior under real-world conditions. By addressing these aspects, the paper can better guide future advancements in material design and processing techniques to enhance the performance and reliability of printed components in various industrial applications.

A critical factor in the adoption of high-speed 3D printing materials is their cost-effectiveness compared to traditional alternatives. While high-speed filaments such as modified PLA, ABS, and PETG offer superior mechanical properties and improved printability, their unit prices are typically higher than conventional thermoplastics due to specialized formulations and enhanced thermal properties. For instance, high-speed PLA can be up to 30% more expensive per kilogram than standard PLA. Additionally, adopting high-speed printing requires investment in upgraded hardware, including printers with advanced motion control systems, reinforced hotends, and optimized cooling mechanisms, which can range from 20% to 50% higher in cost compared to conventional FDM printers. However, the increase in print speed—often exceeding 250 mm/s—leads to a reduction in production time, improving throughput and justifying the initial investment. The return on investment (ROI) depends on the usage volume, with industries engaged in high-throughput manufacturing, such as automotive and aerospace, benefiting the most from accelerated production cycles. Further cost–benefit analyses considering material longevity, waste reduction, and energy efficiency are needed to optimize high-speed material adoption for different applications. Also, as far as compatibility issues are concerned, high-speed thermoplastic materials are compatible with a wide range of FFF/FDM 3D printers without significant issues. While optimized for advanced motion systems, these materials can be effectively used in various printers by adjusting print settings such as extrusion rates, cooling strategies, and motion parameters. Additionally, slicing software profiles help to streamline integration, ensuring smooth printing performance without requiring specialized hardware modifications.

The evolution of high-speed thermoplastics is believed to have a profound impact on industries such as automotive, aerospace, and healthcare, where the need for rapid, reliable, and cost-effective manufacturing is paramount. In the automotive sector, the ability to produce lightweight durable components at high speeds will enable faster prototyping and production cycles, reducing the time to market for new vehicle designs. Moreover, the use of high-speed materials for tooling and fixtures will streamline the assembly processes, increasing operational efficiency. Likewise, in the aerospace industry, the adoption of high-speed materials will support the production of complex lightweight structures that meet stringent performance and safety requirements. High-speed thermoplastics with enhanced thermal and mechanical properties will enable the creation of parts that can withstand the extreme conditions encountered in aerospace applications. Similarly, the healthcare industry will benefit from the ability to quickly produce patient-specific implants, prosthetics, and surgical models using high-speed materials, thereby improving patient outcomes and reducing costs. In this context, as these materials continue to evolve, their adoption will be greatly beneficial across multiple industries.

## 6. Conclusions

The analysis of high-speed 3D printing materials presented in this study highlights their extensive application potential in the field of additive manufacturing. The key findings indicate that these materials significantly outperform traditional thermoplastics in critical areas such as mechanical properties, thermal stability, printability, and performance at high speeds. High-speed PLA, ABS, PETG, and other formulations demonstrate enhanced tensile strength, impact resistance, and flexibility, coupled with superior thermal properties and dimensional stability. Their optimized flow dynamics and rapid crystallization rates allow for consistent high-quality prints at speeds exceeding 250–300 mm/s, addressing the longstanding challenges of warping, poor layer bonding, and surface defects observed in conventional materials. Moreover, their compatibility with advanced post-processing techniques and reduced finishing requirements make them well suited for a wide range of applications across industries, from automotive and aerospace to healthcare and consumer goods.

The potential for high-speed 3D printing materials to reshape the landscape of additive manufacturing is substantial. By enabling faster production cycles without compromising part quality or functionality, these materials bridge the gap between prototyping and full-scale manufacturing. Their ability to meet the growing demands of industries requiring rapid prototyping, on-demand manufacturing, and lightweight yet durable components underscores their role as a key enabler of industrial innovation. Furthermore, the ongoing advancements in high-speed materials are expected to spur the development of new 3D printing technologies, including printers that are optimized for higher extrusion rates, precision cooling, and enhanced accuracy at faster speeds. As high-speed materials continue to evolve, they are likely to unlock new possibilities for customization, multi-material printing, and sustainable manufacturing practices, paving the way for broader adoption of 3D printing in sectors that demand efficiency and precision.

However, achieving the full potential of high-speed materials requires careful con-sideration of the balance between speed, cost, and performance. While these materials of-fer significant advantages, their higher production costs and reliance on specialized equipment may pose barriers to adoption for some users. Ensuring accessibility will require innovations in both material formulation and printer technology to reduce costs while maintaining or enhancing performance. Moreover, understanding the trade-offs involved in material selection—such as balancing mechanical strength with printability or optimizing thermal properties for specific applications—will be critical for leveraging the benefits of high-speed materials effectively. Ultimately, high-speed 3D printing materials represent a vital advancement in additive manufacturing, offering a powerful tool for addressing the complex demands of modern industrial and commercial applications.

## Figures and Tables

**Table 1:** Overview of key thermoplastic materials for 3D printing: properties, advantages, challenges, and the impact of high-speed printing on their use and applications.

Material	Properties	Advantages	Challenges	Impact of High-Speed 3D Printing	Typical Applications
**PLA (Polylactic Acid)**	Low melting point, biodegradable, non-toxic	Easy to print, environmentally friendly, good detail	Limited heat resistance, brittle at low temperatures	High-speed PLA versions reduce cooling times and enhance layer bonding	Prototyping, educational models, decorative items
**ABS (Acrylonitrile Butadiene Styrene)**	High strength, impact-resistant, temperature-resistant	Durable, versatile, suitable for functional parts	Warping issues, emits fumes when printing	High-speed ABS has faster cooling rates and improved thermal stability	Automotive parts, consumer goods, functional prototypes
**PETG (Polyethylene Terephthalate Glycol)**	Strong, impact-resistant, chemically resistant	Better strength than PLA, lower warping than ABS	Can be prone to stringing and oozing	High-speed PETG formulations reduce cooling times and improve adhesion	Packaging, consumer products, medical applications
**PA (Nylon)**	Strong, flexible, abrasion-resistant, moisture-absorbent	High tensile strength, durable, versatile for functional parts	Can absorb moisture, difficult to print without a heated bed	High-speed nylon prints maintain flexibility and strength, reducing warping	Functional prototypes, automotive, robotics
**(PC) Polycarbonate**	High impact resistance, high temperature tolerance	Strong, durable, good for high-performance parts	Difficult to print, requires high temperatures	High-speed polycarbonate has faster extrusion and improved adhesion properties	Aerospace, automotive, engineering parts
**PEEK (Polyetheretherketone)**	Extremely high temperature resistance, strong, chemically resistant	Ideal for aerospace, medical implants, and high-performance applications	Difficult to print, requires high temperature and precision	High-speed PEEK formulations allow for faster print times without compromising strength	Aerospace, medical devices, industrial applications
**Carbon-Fiber-Reinforced Filament**	Strong, lightweight, stiff, high strength-to-weight ratio	Excellent for structural parts requiring stiffness	Can cause excessive wear on nozzles, expensive	Carbon fiber filaments designed for high-speed printing maintain strength while reducing wear	Aerospace, automotive, robotics
**TPU (Thermoplastic Polyurethane)**	Flexible, elastic, wear-resistant, impact-resistant	Suitable for soft, elastomeric parts, good flexibility	Difficult to print at higher speeds, can cause stringing	High-speed TPU formulations offer better flow and faster print times	Footwear, medical devices, consumer products

**Table 2:** Summary of key differences between traditional thermoplastics and high-speed materials across mechanical properties, printability, performance at high speeds, thermal properties, and post-processing requirements.

Category	Traditional Thermoplastics	High-Speed Thermoplastics	Key Improvements
**Mechanical Properties**	Moderate tensile strength, impact resistance, and flexibility	Enhanced tensile strength, better impact resistance, and improved flexibility	Stronger interlayer bonding, reduced internal stresses, and optimized polymer structure
**Printability**	Requires precise settings, prone to warping and stringing at high speeds	Easier to print at high speeds, less prone to defects like warping or stringing	Improved flowability, faster cooling, and reduced thermal expansion
**Performance at High Speeds**	Reduced print quality and structural integrity at high speeds	Maintains quality and mechanical performance at speeds > 250 mm/s	Faster melting, better cooling, and more stable layer adhesion
**Thermal Properties**	Lower heat resistance, limited glass transition temperature (e.g., PLA: 55–65 °C)	Elevated heat resistance and glass transition temperatures (e.g., PLA: up to 80 °C)	Faster crystallization, reduced thermal gradients, and improved thermal stability
**Post-Processing**	Requires extensive sanding or smoothing, structural weaknesses post-finishing	Minimal post-processing needed, improved durability during finishing	Smoother surfaces out of the printer, enhanced compatibility with chemical finishes
